# Managing *Malassezia* species and related infections: new insights into recent natural and synthetic antifungal compounds and their mechanism of action

**DOI:** 10.1007/s11030-026-11526-1

**Published:** 2026-03-29

**Authors:** Damiano Iacovozzi, Simone Carradori, Amar Osmanović, Claudiu T. Supuran, Clemente Capasso, Andrea Angeli

**Affiliations:** 1 Department of Pharmacy, “G. d’Annunzio” University of Chieti-Pescara, 66100 Chieti, Italy; 2 UdA-TechLab, “G. d’Annunzio” University of Chieti-Pescara, 66100 Chieti, Italy; 3 Faculty of Pharmacy, University of Sarajevo, 71000 Sarajevo, Bosnia and Herzegovina; 4 NEUROFARBA Department, Section of Pharmaceutical and Nutraceutical Sciences, University of Florence, 50019 Sesto Fiorentino, Florence, Italy; 5 Department of Biology, Agriculture and Food Sciences, CNR, Institute of Biosciences and Bioresources, Naples, Italy

**Keywords:** *Malassezia*, Carbonic anhydrase, Lipase, Silver nanoparticles, Natural compounds, Probiotics

## Abstract

*Malassezia* spp. are lipophilic yeasts that colonize human and animal skin, being implicated in several dermatological and systemic disorders, including seborrheic dermatitis, pityriasis versicolor, and opportunistic fungemia in immunocompromised individuals. The increasing diffusion of resistant strains and the limited efficacy of conventional azole-based antifungal therapies have prompted the search for alternative treatment strategies. This review explores recent approaches to fighting *Malassezia*, focusing on both natural and synthetic compounds targeting key molecular pathways. Among the most innovative approaches are *Malassezia* carbonic anhydrases, essential enzymes involved in pH regulation and cellular metabolism, which represent promising and selective antifungal targets. The antifungal potential of plant-derived extracts rich in polyphenols, terpenoids, and flavonoids is also discussed, highlighting their ability to interfere with yeast growth and adhesion. Silver nanoparticles are examined as synergistic agents due to their antimicrobial properties and potential as carriers for natural bioactive compounds. Additional perspectives include the use of probiotics/postbiotics to restore skin microbiota balance, enzymes such as chitinase and chitosanase, and the inhibition of lipases, key enzymes in *Malassezia* lipid metabolism. Finally, the growing interest in antimicrobial peptides, both natural and synthetic, opens new avenues for targeted topical formulations. Overall, the integration of natural and synthetic approaches, supported by a deeper understanding of *Malassezia*’s molecular mechanisms, may promote the development of more effective and safer multifactorial therapies.

**Graphical abstract:**

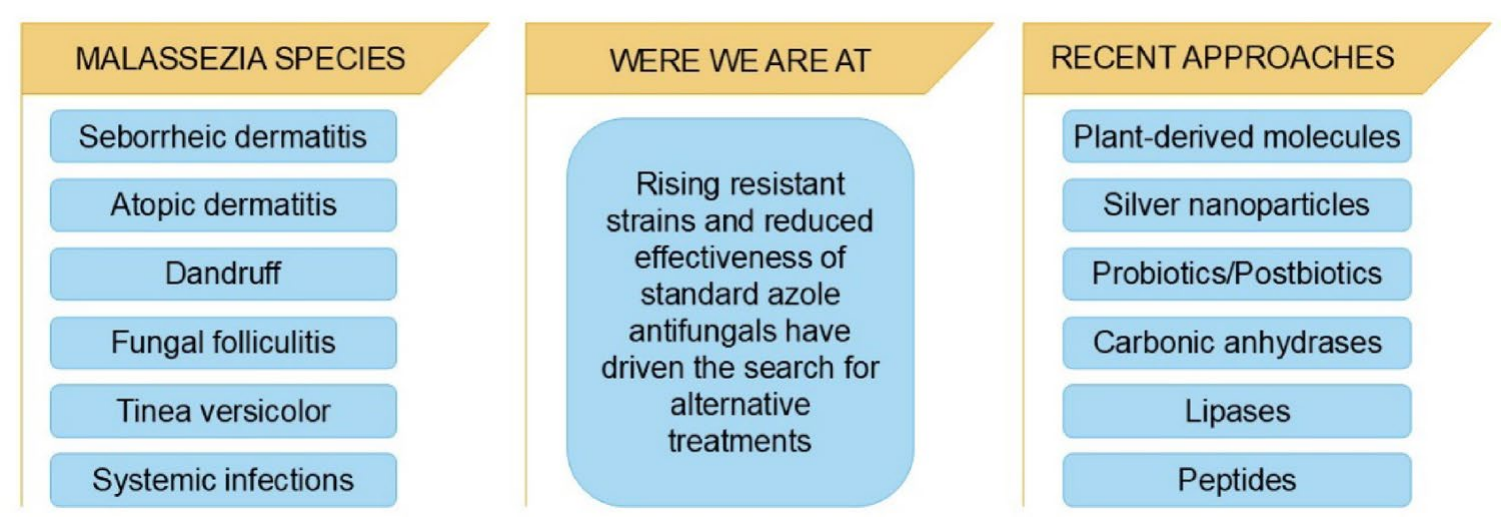

## Introduction

The *Malassezia* genus comprises a distinctive group of lipophilic fungi that have evolved to survive in particularly challenging biological niches such as the skin of humans and other warm-blooded animals [1, 2]. First described in the late nineteenth century by the French scientist Louis-Charles Malassez, these yeasts are now recognized as both commensal residents and opportunistic pathogens. Their dual role has attracted considerable attention in dermatology, microbiology, and immunology due to their relevance in both health and disease [3]. Comparative genomic analyses have revealed that *Malassezia* species possess several genes of bacterial origin, acquired via horizontal gene transfer. These genes are involved in stress tolerance and pathogenic mechanisms, contributing considerably to adaptation and ability to colonize lipid-rich environments [4]. Furthermore, their membrane cells are composed of an unusually high lipid content (15–20%, w/w), higher than that of *Candida albicans* and *Saccharomyces cerevisiae*, which gives them hydrophobicity, increased drug resistance, protects the fungus from the host’s immune responses, and facilitates inflammatory processes [5, 6].

To date, 18 lipid-dependent *Malassezia* species have been identified (Table 1) [7]. Among them, *M. furfur*, *M. sympodialis*, *M. globosa*, and *M. restricta* are the predominant causes of human skin disorders in immunocompetent individuals, while *M. pachydermatis* is associated with dermatitis and otitis in animals. In addition, *M. furfur*, *M. pachydermatis*, and *M. sympodialis* have been linked to systemic infections in immunocompromised patients [1, 8].

**Table 1:** Summaries of taxonomy, occurrence and diseases in humans and animals [1, 9–11]

*Malassezia* species	Humans	Animals
*M. globosa*	Healthy skin; pityriasis versicolor; seborrheic dermatitis; atopic dermatitis	Healthy skin; otitis
*M. dermatitis*	Healthy skin; atopic dermatitis	Not reported
*M. restricta*	Healthy skin; seborrheic dermatitis	
*M. yamatoensis*		
*M. arunalokei*		
*M. japonica*	Healthy skin; seborrheic dermatitis; atopic dermatitis	
*M. obtusa*	Healthy and lesioned skin	
*M. slooffiae*		Healthy skin
*M. furfur*	Healthy skin; pityriasis versicolor; fungemia	
*M. vespertilionis*	Not reported	Healthy skin (bat)
*M. psittaci*		Healthy skin (parrot)
*M. brasiliensis*		
*M. caprae*		Healthy skin (goat)
*M. cuniculi*		Healthy skin (rabbit)
*M. pachydermatis*	Healthy skin, fungemia	Healthy and lesioned skin
*M. equina*	Not reported	
*M. sympodialis*	Healthy skin, atopic dermatitis; seborrheic dermatitis	Healthy skin; otitis
*M. nana*	Not reported	

A defining biological trait of *Malassezia* is its strict lipid dependence. Unlike most fungi, which can synthesize their own fatty acids, *Malassezia* requires exogenous lipids for survival and proliferation. This dependence allows it to thrive on the human skin surface, where sebum provides a rich supply of triglycerides and other lipids crucial for its growth. The skin serves as the primary biological niche for *Malassezia*, focusing on regions with dense sebaceous glands, such as the scalp, face, and upper torso, and to a lesser extent on the feet [11]. Species distribution across body sites is not uniform: *M. restricta* predominates in the auditory canal, glabella, and retroauricular area, whereas *M. globosa* is abundant on the back, occiput, and inguinal folds. Other species occur less frequently and exhibit broader but less specific distribution patterns [10]. These sebaceous environments, characterized by moderate temperature, high humidity, and lipid abundance, provide ideal conditions for *Malassezia* colonization. In healthy individuals, *Malassezia* functions as a commensal organism and is a dominant component of the cutaneous mycobiome [12]. The skin microbiota, comprising bacteria, fungi, viruses, and other microorganisms, forms a complex and dynamic ecosystem that interacts with the host to maintain skin homeostasis. Colonization by *Malassezia* begins shortly after birth, facilitated by maternal contact, environmental exposure, and caregiver interactions [12]. Although typically a harmless commensal, *Malassezia* can become pathogenic under specific conditions such as immune suppression, skin barrier disruption, or microbiome imbalance. This transition is associated with disorders including dandruff, seborrheic dermatitis, *Malassezia* folliculitis, and atopic dermatitis [13, 14]. In severe cases, especially in neonates and immunocompromised patients, *Malassezia* may cause systemic infections, underscoring its opportunistic potential [15]. The pathogenic mechanisms of *Malassezia* involve the secretion of hydrolytic enzymes such as lipases, phospholipases, and proteases, which degrade skin lipids and proteins to provide nutrients and promote tissue invasion. Furthermore, *Malassezia* can modulate host immunity through the production of bioactive molecules that trigger cytokine and chemokine release, influencing local inflammatory responses [16]. The different species of *Malassezia* have distinct colonization niches and pathological associations that vary according to geographical region and population. *M. globosa* is most frequently implicated in human skin disorders such as pityriasis versicolor and dandruff/seborrheic dermatitis, while *M. restricta* primarily colonizes the scalp and facial areas and is linked to seborrheic and atopic dermatitis [17, 18]. Less common species, including *M. sympodialis* and *M. dermatis*, have been detected on both healthy and diseased skin, with occasional involvement in systemic infections among immunocompromised patients [19]. In animals, *M. pachydermatis* is a major cause of otitis and dermatitis and may act as a zoonotic pathogen, transferring from pets to humans and, in rare cases, causing bloodstream infections [20].

**Fig. 1:**
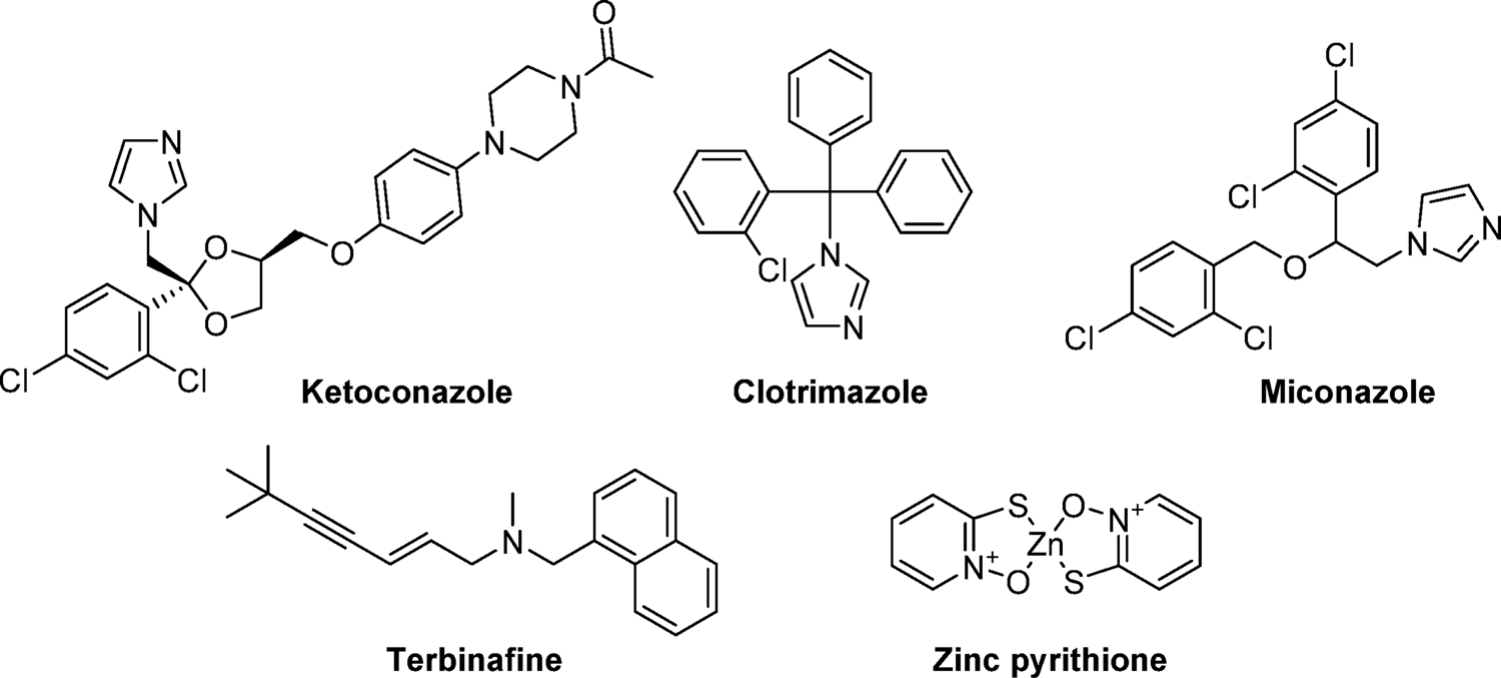
Structures of drugs currently used against *Malassezia* species

The treatment of *Malassezia*-associated conditions is complicated by yeast lipid dependence, biofilm formation, and resistant cell wall, which increase the tolerance to antifungal agents. Topical therapies, mainly azoles such as ketoconazole, clotrimazole, miconazole and allylamines (terbinafine), remain the primary treatment options for superficial infections (Fig. 1) [21, 22].

Non-azole compounds such as selenium sulfide (Se_2_S) and zinc pyrithione, commonly used in medicated shampoos, also exhibit antifungal activity and are effective adjuncts for managing dandruff and seborrheic dermatitis [23]. Table 2 summarizes main and current approaches for managing *Malassezia* species.

**Table 2:** Active ingredients found in commercial products for managing *Malassezia*-related diseases

Topical or systemic agents			
Ketoconazole	Nizoral®, Extina®	Shampoo, cream, foam, gel	[24]
Miconazole	Daktarin®, Conofite®	Cream, spray, powder	[25, 26]
Clotrimazole	Canesten®		[27, 28]
Sertaconazole	Ertaczo®, Dermofix®	Cream	[29, 30]
Selenium sulfide	Vichy Dercos® DS	Shampoo	[31–34]
Ciclopirox olamine	Sebiprox®, Deltacrin® DS		[35–37]
Itraconazole	Sporanox®	Capsule, oral solution	[38, 39]
Fluconazole	Diflucan®	Oral suspension	[38, 40]
Terbinafine	Lamisil®	Tablets, cream	[41–43]
Butenafine	Lotrimin Ultra®	Cream	[44, 45]
Adjunctive agents			
Salicylic acid	Deltacrin® DS	Shampoo, lotion	[46–48]
Zinc pyrithione	Head & Shoulders® Classic Clean	Shampoo	[49, 50]
Acetic/boric acid	MalAcetic Aural®	Solution	[51–53]
Chlorhexidine	Clorexyderm®	Foam	[54]

Currently, azole antifungals are the recommended treatment for *Malassezia*-related diseases. Azoles, such as ketoconazole (topical) or fluconazole (systemic), work by inhibiting lanosterol 14α-demethylase. This enzyme helps conversion of lanosterol into ergosterol, a mycosterol and essential component of fungal cell membranes. Ergosterol is not produced by the human body, therefore the mechanism of action of azoles is highly selective. The disruption of ergosterol synthesis in the fungal cell membrane causes membrane instability, leading to the leakage of cellular components and, ultimately, the fungal cell’s death (fungicidal effect) or the inhibition of fungal growth (fungistatic effect). Azoles are frequently applied topically as a shampoo or cream to treat tinea versicolor and seborrheic dermatitis. On the other hand, more severe cases of *Malassezia* folliculitis should be treated with oral fluconazole or itraconazole [23, 55]. Terbinafine and other allylamines block the squalene epoxidase enzyme, halting the synthesis of ergosterol by preventing squalene from being converted to 2,3-oxidosqualene. The fungal cell membrane becomes weaker when ergosterol is lacking and squalene builds up. Cellular contents leak out because of this weakening, which increases membrane permeability. Terbinafine shows significant efficacy against some bacterial species, such as *M. sympodialis*, and is commonly used as a topical formulation to treat superficial infections [23, 56]. Selenium sulfide has been shown to slow down the rate of cell turnover in the epidermis, which reduces the number of *Malassezia* organisms that live on the skin’s surface, even though the exact mechanisms by which it produces its antifungal effects are still unclear. It is an essential ingredient in anti-dandruff shampoo formulations [23, 57]. Ciclopirox disrupts vital intracellular functions, by preventing the transport of components required for cell division. It has been shown that the substance in question prevents the synthesis of protein, ribonucleic acid, and deoxyribonucleic acid. Cell death results from this disruption’s direct impact on cellular metabolism [23, 58]. Salicylic acid is primarily used as a keratolytic and lipophilic agent to treat conditions related to *Malassezia*, such as folliculitis, seborrheic dermatitis, and dandruff. Salicylic acid works by breaking intercellular cohesive bonds in the stratum corneum, which causes flaky, infected skin to shed. Furthermore, because the agent is lipophilic, it can enter sebaceous glands, decrease oiliness and limit the yeast’s food source [59]. Zinc pyrithione is an antifungal agent that causes severe toxic stress, mitochondrial dysfunction, and the inhibition of iron-sulfur cluster proteins by raising cellular copper and zinc levels. Fungal metabolism is disrupted by the metal toxicity, which lowers the expression of lipase, essential for survival and growth rate [23, 60]. The synergistic, multimodal action of the combination of acetic and boric acid, commonly used in veterinary otic solutions and wipes, creates an unfriendly environment for the yeast and physically destroys its structure. Because *Malassezia* prefers alkaline and lipid-rich environments, this combination works especially well [53]. Finally, chlorhexidine acts as a broad-spectrum, cationic, biguanide antiseptic to treat *Malassezia* infections, which are most frequently seen in humans (dandruff/seborrheic dermatitis) and dogs (dermatitis). Chlorhexidine compromises the structural integrity of the yeast cell wall and membrane. When combined with other agents, like cetrimide, or added to shampoos that contain miconazole, the efficacy of chlorhexidine is increased [61]. Despite the effectiveness of current therapies, increasing antifungal resistance and limited systemic treatment options highlight the need for novel therapeutic strategies.

## Literature survey for the treatment of *Malassezia* species

This work is a narrative review. The literature search was conducted in February 2026 searching in two official scientific databases, PubMed and Scopus, and using combination of the keywords “*Malassezia*”, “antifungal”, and “anti-dandruff”. The search was limited to articles, book chapters, and reviews spanning from 2020 to 2025, without applying additional filters. Approximately 580 records were identified, and 467 documents were selected for further screening. To ensure a comprehensive and unbiased selection, all retrieved records were screened regardless of the *Malassezia* species discussed, and studies were included based on relevance to antifungal activity. Selected papers were then categorized thematically to provide a clear, species-independent summary of: (i) in vitro and in vivo studies evaluating antifungal activity; (ii) clinical and observational data on anti-dandruff interventions; (iii) comparative assessments of compounds, active ingredients, and formulations.

### Bioactive components and extracts of plants

Resistance to current drug options (such as ketoconazole, zinc pyrithione, or selenium sulfide) against *Malassezia* species and consumer preference for “natural” alternatives renewed interest in plant-derived bioactive compounds as sources of therapeutic agents. Plants offer structurally diverse secondary metabolites (like flavonoids, tannins, terpenoids, alkaloids, and saponins) that exhibit antifungal activity through multiple mechanisms: disruption of cell membranes, inhibition of ergosterol synthesis, interference with biofilm formation, and inhibition of fungal enzymes such as lipases and proteases [62]. Also, natural bioactive compounds base their ethnopharmacological relevance on antioxidant and anti-inflammatory properties, as well as activity against drug-resistant and drug-sensible microbial strains [63]. Extracts may also display anti-inflammatory and antioxidant properties that mitigate host responses to *Malassezia*-driven irritation. Importantly, synergistic interactions among components within the whole phytocomplex can enhance efficacy and reduce the likelihood of resistance compared with single-target drugs [64]. Taken together, these characteristics highlight plant-derived metabolites as particularly appealing candidates for managing *Malassezia*-associated conditions, where multi-target mechanisms and low resistance potential may offer advantages over conventional antifungals.

**Fig. 2:**
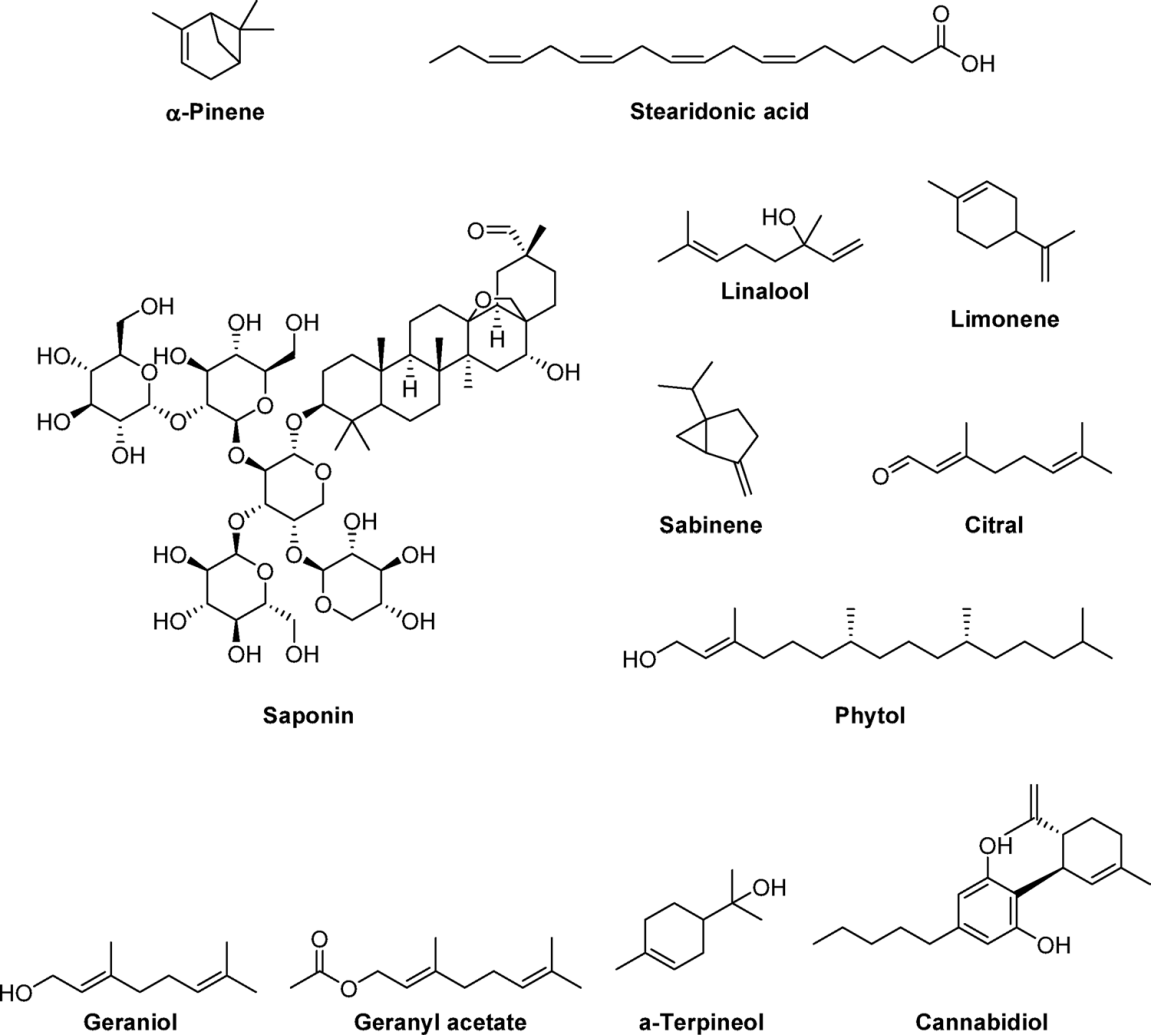
Natural products evaluated for the treatment of *Malassezia* species

The essential oils from different cultivars of *Boswellia sacra* Flück., extracted from the frankincense (an aromatic oleo-gum resin), were comparatively evaluated for their effects against *Malassezia furfur*. The essential oils were obtained through successive hydrodistillations over 2, 4, and 6 h. All grades of essential oils varied in composition and the main analytical components were sesquiterpenes, diterpenes, and monoterpenes. Each hydrodistillation step resulted in the reduction of monoterpenes and in the increase of sesquiterpenes. The primary analyte after 2 h of hydrodistillation was α-pinene (71.09–79.59%) (Fig. 2). Antifungal activity against *M. furfur* showed minimum inhibitory concentration (MIC) values inferior than 0.270 mg/mL [65]. While the study did not prove that α-pinene is the main responsible for antifungal activity, other studies have already investigated and highlighted the role of α-pinene against fungi [66].

Similarly, Kumari et al. investigated the antifungal activity of essential oils of *Curcuma longa* L., *Tagetes minuta* L., *Matricaria chamomilla* L., *Salvia sclarea* L., *Ocimum sanctum* L., *Ocimum basilicum* L., and *Cymbopogon martini* (Roxb.) Will. Watson. The essential oil of *C. martini* (commonly named palmarosa) demonstrated the best antifungal activity against *Malassezia furfur*, with 29.4 ± 2.70 mm inhibition zone, MIC = 0.27 mg/mL, and minimum fungicidal concentration (MFC) = 1.1 mg/mL. At 2 × MIC, the essential oil of *C. martini* inhibited the growth of *M. furfur* by 88.36% within 4 h. Investigations into the cell ultrastructure of *M. furfur* treated with *C. martini* oil showed disruption of the cell surface by downregulation of efflux pumps. However, no interference with membrane precursors was observed. Moreover, *C. martini* oil increased the production of intracellular reactive oxygen species, causing degradation of crude proteins but no fragmentation of DNA. GC/MS found geraniol and geranyl acetate (68.46% and 10.27%, respectively) (Fig. 2) as the predominant analytes in *C. martini* oil [67].

Fatty acids from chloroform and ethanol extracts of flowers of *Senna auriculata* (L.) Roxb. and *Senna alata* (Linnaeus) Roxburgh were evaluated against *Malassezia furfur* at different concentrations. Antifungal activity was assayed through measurement of inhibition zones. Chloroform extract of *S. auriculata* exhibited better antifungal activity than *S. alata*, with a dose-dependent trend (3.3 mm at 80 μg/mL and 6.7 mm at 160 μg/mL). Conversely, ethanol extract of *S. alata* exhibited better antifungal activity than *S. auriculata* (4.3 mm at 80 μg/mL and 7.7 at 160 μg/mL). Ketoconazole was used as reference drug for comparison (5.9 ± 0.74 mm at 30 μg/mL). GC–MS analysis revealed the presence of fatty acids such as stearic acid, palmitic acid, and *i*-propyl 11,12-methylene-octadecanoate, as well as fatty alcohols such as 1-tetracosanol and 1-hexadecanol [68]. The antifungal activity of fatty acids is documented in earlier studies [69]. However, further validation is needed to establish the role of fatty acids from the *Senna* genus and to distinguish the activity of fatty acids from the activity of other bioactive compounds found in the extracts. In a subsequent study, the focus shifted on the leaves of *Senna alata*. The analytes found in the *n*-hexane extract were identified being stearidonic acid (Fig. 2), trichosanic acid, and 9-ene-methyl palmitate the main compounds. These, along with ketoconazole as a reference, were studied with molecular docking against the fungal target lanosterol 14α-demethylase (CYP51). Because the three-dimensional structure of *Malassezia* lanosterol 14α-demethylase has not yet been experimentally resolved and no co-crystal structure is available in the Protein Data Bank, the authors first constructed a structural model of the enzyme using homology modeling. The resulting model was subsequently used as the receptor for docking calculations. Stearidonic acid and trichosanic acid demonstrated better binding affinity at -7.2 kcal/mol, forming hydrogen bonds with Ser329 and Ser456, respectively, and additional Pi-Sigma and Pi-Alkyl interactions within the active site cavity. Although the reference antifungal drug ketoconazole displayed slightly stronger binding (-7.9 kcal/mol), the docking results suggested that fatty acid components present in the extract may contribute to the observed antifungal activity through potential interaction with CYP51 and disruption of ergosterol biosynthesis. Antifungal activity of the *n*-hexane extract showed dose-dependent inhibition zones (29.89 mm at the minimum tested concentration of 50 mg/mL), whereas ketoconazole was used as reference drug (19.44 mm at 20 mg/mL) [70].

Choudhary et al. evaluated the antifungal activity of the seed waste surfactants from *Carthamus tinctorius* L. (commonly named safflower) against *Malassezia furfur*. Saponin (Fig. 2) was the main analyte isolated following cold maceration and aqueous methanol extraction. The extract showed antifungal activity with a dose dependent trend ranging from 0.47 ± 0.06 mg/mL to 0.11 ± 0.00 mg/mL. Disk diffusion showed 15.6 mm zone of inhibition at 1 mg/mL [71].

Liao et al. evaluated the essential oil from *Zanthoxylum schinifolium* Siebold & Zucc. for antifungal activity against *Malassezia restricta*. *Z. schinifolium* is a native Chinese flowering plant member of the Rutaceae family. The essential oil contained linalool, limonene, and sabinene (Fig. 2) as major analytes (34.15%, 21.24%, and 10.39%, respectively). The essential oil exhibited values of MFC and MIC equal to 10 mg/mL and 2.5 mg/mL, respectively. Linalool showed MIC value equal to 2.5 mg/mL, while limonene did not show any significant activity at the tested concentrations (MIC > 20 mg/mL). Other minor compounds in the essential oil showed significant MIC values, such as terpinolene (MIC = 2.5 mg/mL). Liao and colleagues suggested that the activity of different compounds may play a synergistic role in antifungal activity, especially fungicidal activity. At 1 × MIC and 2 × MIC, the essential oil altered the permeability and induced the destruction of cell membranes, leading to cellular leakage. Moreover, the essential oil caused oxidative stress in respiratory metabolism and induced modifications in cell morphology, leading to the death of *M. restricta* [72].

Citral (Fig. 2) is a monoterpene commonly found in the essential oil of several plants, such as *Backhousia citriodora* F. Muell., *Melissa officinalis* L., and *Cymbopogon citratus* (DC. ex Nees) Stapf. Liu et al. investigated the antifungal activity of citral on *Malassezia furfur*. MIC and MFC values were 200 ± 15 μg/mL and 300 ± 25 μg/mL, respectively. Ketoconazole, used as reference drug, showed MIC and MFC equal to 0.13 ± 0.05 μg/mL and 0.25 ± 0.09 μg/mL, respectively. Notably, citral increased the number of yeast cells possessing mycelial forms (2.6-fold increase). Biological investigations showed that citral induced externalization of phosphatidylserine and membrane depolarization. Moreover, activation of metacaspase and fragmentation of DNA were observed to be related to citral-induced apoptosis on *M. furfur*. Cell viability on HaCaT cells demonstrated the absence of cytotoxicity at all concentrations evaluated. Citral also reduced adhesion (about 40% reduction after 24 h at 25 mg/mL at 1:20 HaCaT cells to yeasts ratio) and invasiveness (about 30% reduction) of *M. furfur* into HaCaT cells with a dose-dependent trend. Lastly, in preliminary studies, citral downregulated the expression of toll-like receptor 2 (TLR2) in HaCaT cells infected with *M. furfur*, and increased human beta-defensin-2 (HBD-2) [73].

Gebremariam et al. evaluated the antifungal activity of leaf gel and leaf latex of *Aloe adigratana* Reynolds, a succulent shrub native to Ethiopia and Eritrea. Clinical specimens of *Malassezia furfur*, *Malassezia globosa*, and *Malassezia restricta* were collected by volunteers with dandruff. Disk diffusion test revealed that the leaf latex of *A. adigratana* exhibited dose-dependent inhibition and was more effective on *M. globosa* (19.33 ± 0.58 mm at 250 mg/mL compared to 19.27 ± 0.25 mm at 50 μg/mL). The leaf gel showed a similar trend. MFC/MIC ratio equal to 2.0 implied fungicidal activity and confirmed the highest activity of both leaf gel and latex on *M. globosa* (MIC = 0.24 ± 0.00 mg/mL for latex extract and MIC = 0.48 ± 0.00 mg/mL for gel extract) [74]. The study did not address the chemical profile of leaf latex, while the profile of leaf gel was evaluated in a previous study and showed phytol (Fig. 2) as the main compound (26.38%) [75]. However, GC–MS analysis showed the phytochemical profile of the essential oil from *A. adigratana*. The most abundant analyte was still phytol (33.78%) [74]. Further studies are needed to identify the main compounds responsible for the inhibition of *Malassezia* species. Nevertheless, phytol seems a potential candidate and, in fact, a few studies have explored the antifungal activity of phytol [76–78].

The combination of leaf extract from *Azadirachta indica* A. Juss. (commonly known as neem) and herbal extract from *Salvia rosmarinus* Spenn (commonly known as rosemary) demonstrated antifungal activity against *Malassezia furfur*. Disk diffusion method showed that the activity of the 2:1 combination of neem and rosemary was aligned with the activity of ketoconazole, used as reference drug (21 mm against 24 mm, respectively). Minimum inhibitory concentration assay reported the MIC value to be 12.5 mg/mL. The 2:1 combination was formulated to prepare both leave‐in hair tonic and herbal gel. Both formulations were evaluated on mice by Franz diffusion cells to assess ex vivo skin deposition of rosmarinic acid and rutin, the two main analytes identified by UPLC. Results showed a significant deposition in the epidermis layer, where *M. furfur* is usually found [79].

Zagórska-Dziok et al. developed a new hydrogel incorporating α-terpineol (Fig. 2) as a penetration enhancer and cannabidiol (Fig. 2) as the main potential therapeutic agent. They evaluated the hydrogel on *Malassezia furfur* and reported that the hydrogel loaded with 5% α-terpineol and cannabidiol exhibited 16 mm diameter of inhibition, compared to 11 mm of the formulation at 1% [80].

Ahmad et al. investigated the aqueous and ethanol extracts of rhizomes from *Zingiber officinale* Roscoe (commonly known as ginger) for antifungal activity against *Malassezia furfur*. Disk diffusion method showed dose-dependent trend for both extracts, with the ethanol extract demonstrating better activity than the aqueous one. 200 mg/mL of ethanolic extract resulted in 19.4 mm inhibition zone, compared to 12.8 mg/mL of aqueous extract and 20.7 mm of fluconazole at 40 mg/mL, used as a reference drug. GC/MS analysis also described the phytochemical composition of ethanolic extract [81].

It is important to acknowledge that the chemical variability of plant extracts, the absence of standardized preparations, and the limited availability of structure–activity relationship analyses significantly complicate translational development. Optimal harvest time, cultivar differences, growing methods, and farming techniques affect the chemical variability of plant extracts. Multidimensional analysis is rising as a practical approach to assess quality differences related to chemical components. The combination of instrumental analyses (NMR, GC–MS, HPLC), principal component analysis (PCA), and chemometrics allows us to create a database of patterns or chemical fingerprints, which can be used to compare chemical differences from a qualitative and quantitative perspective. For example, this approach (similar to fingerprint-based docking between ligands and proteins [82, 83]) has been successfully used in the quantitative profiling of pomegranates [84] and *Lavandula* cultivars [85], and the qualitative profiling of apple aroma [86]. Regardless of standardization, future investigations should focus on assessing efficacy in physiologically relevant models to determine whether these natural compounds can realistically advance toward therapeutic applications for *Malassezia*-associated disorders.

### Silver nanoparticles

Silver nanoparticles have attracted attention as broad-spectrum antimicrobial agents with potential application in anti-dandruff formulations targeting *Malassezia* species. The antimicrobial activity of silver nanoparticles arises from multiple concurrent mechanisms: release of Ag^+^ ions that interact with thiol groups in proteins, generation of reactive oxygen species, disruption of cell membranes, and interference with DNA replication and cellular respiration. These multimodal actions reduce the likelihood of rapid resistance development compared with single-target antifungals. Silver nanoparticles offer several attractive features: efficacy at low concentrations due to high surface-area-to-volume ratio, ability to act in lipid-rich environments when appropriately surface-modified, and potential synergism with conventional antifungals to lower required doses [87]. Moreover, biogenic silver nanoparticles synthesized using plant extracts provide added advantages through capping agents that can enhance stability, modulate lipophilicity, and contribute complementary antifungal or anti-inflammatory effects [88]. Specifically, phytochemicals serve as natural stabilizers by adsorbing onto the surface of nanoparticles to provide steric hindrance and prevent agglomeration. This role explicitly aligns with green chemistry principles, such as using renewable feedstocks, preventing waste, and designing safer chemicals [89]. Biogenic agents are biodegradable and biocompatible, are suitable for living systems without producing hazardous residues, and can replace commonly used synthetic capping agents, such as cetyltrimethylammonium bromide (CTAB), sodium dodecyl sulfate (SDS), or polyethylene glycol (PEG), which are often nonbiodegradable and toxic [90]. Examples include aucubin, an iridoid glycoside from asterids, which spontaneously capped zinc nanoparticles through hydrogen bonding [91]; hydroalcoholic dandelion flower extract, which reduced AgNO_3_ during the synthesis of silver nanoparticles [92]; or green tea leaf and olive extracts, similarly involved in the bio-reduction of silver ions to silver nanoparticles [93].

Silver nanoparticles obtained from the OF1 strain of *Streptomyces xinghaiensis*, a strain of actinomycetota, exhibited antifungal activity against *Malassezia furfur* [94]. The minimum inhibitory concentration value was 32 μg/mL, whereas 4 μg/mL for fluconazole. The minimum inhibitory concentration and minimum fungicidal concentration values for *M. furfur* were 32 μg/mL and 48 μg/mL, respectively, against 4 and μg/mL and 96 μg/mL for fluconazole. Both amphotericin B and ketoconazole did not exhibit inhibitory or fungicidal activity (> 1024 μg/mL) in the range of tested concentrations. The combination of nanoparticles with fluconazole or amphotericin B exhibited additive or indifferent activity, with 1.0 value of fractional inhibitory concentration (FIC) index [95]. Moreover, combination of nanoparticles and antibiotics at FIC concentrations reduced the overall in vitro cytotoxicity on HeLa cells and mouse embryonic fibroblasts 3T3.

Mussin et al. evaluated silver nanoparticles against Malassezia furfur strains isolated from human samples of clinical diagnosis such as atopic dermatitis, seborrheic dermatitis, and pityriasis versicolor. Antifungal activity showed MFC/MIC ratios less than 4 for all strains, implying a fungicidal activity rather than fungistatic. Then, the synergistic combination of nanoparticles and ketoconazole was evaluated using a gel formulation based on polyacrylic acid polymers (Carbopol® 940). The increase in diameter of inhibition zones showed a moderate increase in antifungal activity of the gel (28 mm) compared to nanoparticles and ketoconazole alone (13 mm and 24 mm, respectively) [96].

In a subsequent study, Mussin et al. explored the role of *Acanthospermum australe* (Loefl.) Kuntze in preparing silver nanoparticles, and evaluated cytotoxic and antifungal activities on *Malassezia furfur*, *Malassezia globosa*, *Malassezia sympodialis*, and *Malassezia restricta*. Cytotoxicity assay on peripheral blood mononuclear cells revealed the effects of the aqueous extract of *A. australe* in mitigating the cytotoxic effects of silver. Metabolites from *A. australe* reduced the overall surface area and the active interacting sites of silver nanoparticles. Initially, the coating provided by the phytocompounds showed cell viability values below the cut-off of 70% after 24 h; eventually, values grew above the cut-off after 72 h. Minimum fungicidal concentration values showed an overall inhibition of all species, with *M. globosa* being the most affected (0.125 μg/mL). MFC/MIC ratio less than 4 suggested fungicidal activity. However, the antifungal effect was attributed to silver ions only, while the plant extract contributed to the stability of nanoparticles possibly by reducing silver ions to silver nanoparticles [97].

In their latest study, Mussin and Giusiano continued the investigation on *Acanthospermum australe* (Loefl.) Kuntze by characterizing the phytocomplex of the essential oil through gas chromatography-mass spectrometry. The main analytes were germacrene A, γ-cadinene, and *trans*-caryophyllene, representing over 60% of the total. The essential oil did not exhibit cytotoxicity on peripheral blood mononuclear cells. Then, the synergistic activity of essential oil from *A. australe* leaves and silver nanoparticles from aqueous extract of *A. australe* was assessed. Only *M. globosa* showed meaningful results with a fractional inhibitory concentration index of 0.5, indicating synergism. Notably, the reduction in MIC fold decrease (4 times reduction) of both silver nanoparticles and essential oil validated the synergistic effect and improved the overall antifungal activity [98].

### Probiotics and postbiotics

Probiotics are “live microorganisms that, when administered in adequate amounts, confer a health benefit on the host” [99]. Postbiotics are “preparation of inanimate microorganisms and/or their components that confers a health benefit on the host” [100]. The antifungal effects arise from multiple mechanisms: competitive exclusion for adhesion sites and nutrients, production of antimicrobial metabolites (organic acids, hydrogen peroxide, bacteriocins), modulation of local pH, and stimulation of host immune responses that enhance fungal clearance. These multifaceted actions can suppress pathogenic fungi directly and indirectly while promoting a balanced microbiota [101]. Against cutaneous and mucosal fungi, including *Candida* and filamentous species, selected probiotic strains (notably *Lactobacillus*, *Bifidobacterium*, and certain *Bacillus* spp.) have shown inhibitory activity in vitro and protective effects in animal and clinical studies [102]. Probiotic interventions are also gaining increasing attention in the context of *Malassezia*-associated conditions, as several strains can modulate skin microbiota and local immune responses in ways that may limit fungal overgrowth. Although clinical evidence is still limited, early findings suggest that probiotics may serve as useful adjunctive or complementary strategies in the management of dandruff and seborrheic dermatitis, warranting further investigation. Two strains of *Lactiplantibacillus plantarum* derived from leaves of green tea (*Camellia sinensis* (L.) Kuntze) demonstrated in vitro antifungal activity against *Malassezia restricta* and *Malassezia globosa*. Both APsulloc 331,266 and APsulloc 331,261 strains decreased the growth of *M. restricta* and *M. globosa* at 1:1 and 1:0.1 ratios (v/v). Moreover, the conditioned media derived from the two strains inhibited the growth of *M. restricta* and *M. globosa* in a dose-dependent trend (almost no growth at 100% of conditioned medium). Gene analysis by PCR and electrophoresis revealed the presence of *pln* loci in *L. plantarum* strains, responsible for encoding plantaricin antimicrobial peptides. Plantaricins, class II bacteriocins, may be responsible for the antifungal activity, but further studies are required to validate the role [103].

Meng et al. evaluated the in vitro activity of supernatants and pellets, obtained from commercial probiotic strains, on *Malassezia furfur*. They found that *Lacticaseibacillus rhamnosus* and *Bifidobacterium animalis* subsp. *lactis* exhibited antifungal activity. Supernatant from *B. lactis* strains HN019, B420, and Bi07 exhibited over 90% of the inhibition of *M. furfur* within 48 h, and its effect remained consistent up to 96 h. Similarly, supernatant from *L. rhamnosus* HN001 exhibited 90% inhibition within 48 h, but its effect decreased to 60% over 72 h. Pellets demonstrated significant activity, too. Consistently with the supernatant, pellets from HN019 achieved 90% inhibition within 48 h, whereas pellets from Bi07 and B420 achieved about 60%. The inhibition rate of all pellets decreased below 20% after 72 h. No further analyses were conducted to determine the nature of proteins in pellets. However, investigations into organic acids found in supernatants showed that mixtures of lactic acid, butyric acid, propionic acid, and acetic acid (derived from probiotic strains) could achieve inhibition up to 96% at 72 h [104].

### Malassezia carbonic anhydrases as innovative targets

Carbonic anhydrases (CAs) are zinc-containing metalloenzymes that catalyze the reversible hydration of carbon dioxide to bicarbonate and protons, playing central roles in pH regulation, CO_2_/bicarbonate homeostasis, and metabolic processes across organisms. In yeasts of the *Malassezia* genus, β-class CAs represent attractive antifungal targets because β-CAs are absent in humans, who exclusively express α-class isoforms. This fundamental phylogenetic divergence provides a strong rationale for selective therapeutic intervention that has been already explored and reviewed [4, 105].

**Fig. 3:**
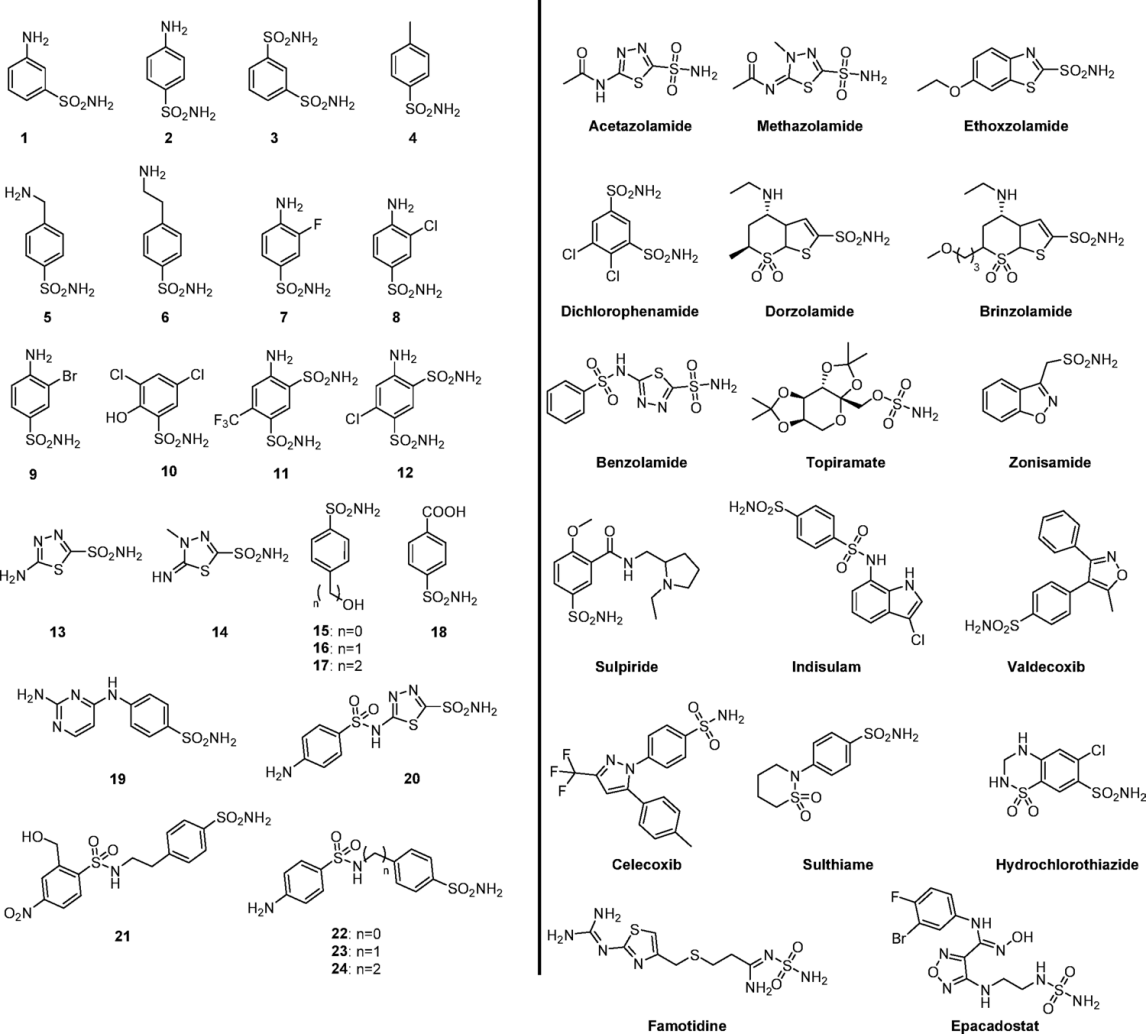
Structures of primary sulfonamide derivatives as carbonic anhydrase inhibitors [106, 107]

A range of primary sulfonamide derivatives, previously recognized as CA inhibitors, has been evaluated against β-CAs from *Malassezia* species [106, 107]. Across studies, several clinically used sulfonamides, including acetazolamide, brinzolamide, valdecoxib, sulthiame, and indisulam (Fig. 3) displayed submicromolar inhibition constants toward specific β-CAs.

**Fig. 4:**
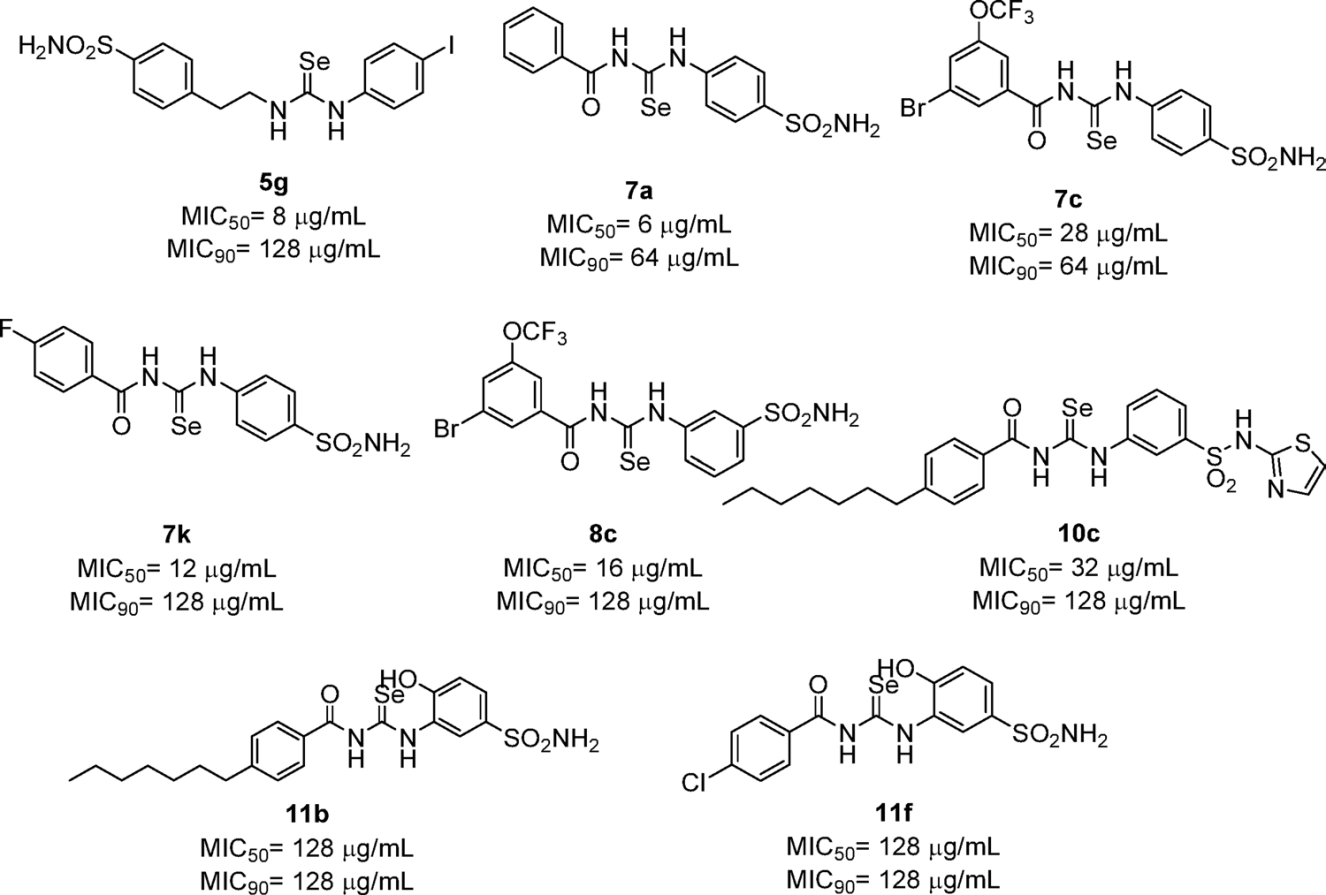
Sulfonamide-containing selenoureido compounds evaluated as carbonic anhydrase inhibitors [110]

However, marked species-dependent variability was consistently observed. The inhibition profiles of β-CAs from *M. globosa*, *M. restricta*, and *M. pachydermatis* differed substantially, indicating significant structural or microenvironmental differences within their active sites despite conservation of the canonical β-CA zinc-binding motif [107–109]. These findings collectively suggest that β-CAs from different *Malassezia* species cannot be considered pharmacologically interchangeable. Although many sulfonamides exhibited potent enzyme inhibition in vitro, translation into antifungal activity in cell-based assays was often limited, with relatively high MIC values. This discrepancy likely reflects restricted cellular permeability and emphasizes the importance of physicochemical optimization beyond enzymatic potency alone. Nevertheless, selected compounds demonstrated improved in vivo efficacy. Notably, compound 6 reduced fungal proliferation in a murine model of *Malassezia*-associated dandruff, achieving antidandruff activity comparable to ketoconazole, thereby validating β-CA inhibition as a viable antifungal strategy under physiological conditions. Subsequent structural refinement led to the development of selenoureido and acylselenoureido sulfonamide derivatives (Fig. 4), which demonstrated enhanced antifungal activity, particularly against *M. pachydermatis* [110]. Their mechanism appears to combine β-CA inhibition with selenium-mediated oxidative stress, resulting in a dual antifungal effect. Structural optimization highlighted the critical role of selenium incorporation, as sulfur or oxygen substitution markedly reduced potency. Importantly, several derivatives showed low cytotoxicity toward mammalian cell lines and minimal hemolytic activity, supporting their therapeutic potential. In some cases, antifungal efficacy approached or exceeded that of reference agents such as ketoconazole or selenium disulfide, particularly against veterinary isolates of *M. pachydermatis* [111].

**Fig. 5:**
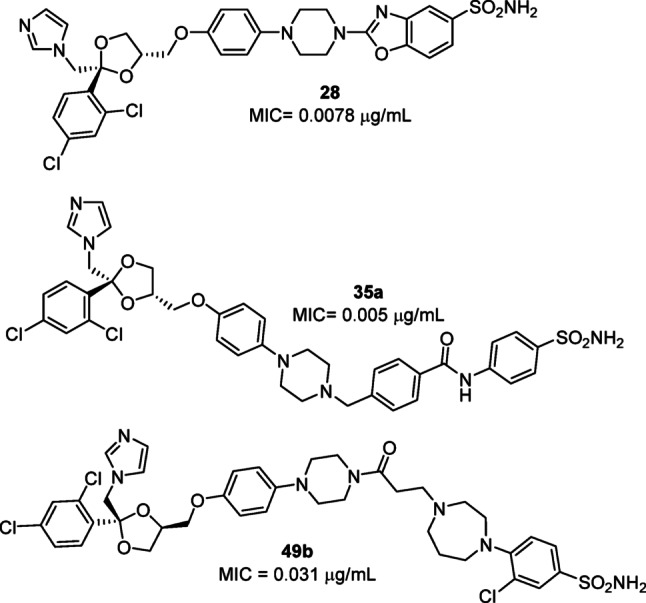
Structures of selected ketoconazole-based derivatives reported by Renzi et al. [112]

A complementary dual-target strategy has also been explored through ketoconazole-based hybrids bearing benzenesulfonamide moieties (Fig. 5) [112]. These compounds were designed to inhibit both lanosterol-14α-demethylase (CYP51) and fungal β-CAs. The resulting derivatives maintained CYP51 inhibition while achieving submicromolar activity against *Malassezia* β-CAs, especially from *M. pachydermatis* and *M. restricta*. This multitarget approach may enhance antifungal efficacy and mitigate resistance development, while reducing cytotoxic liabilities.

Beyond primary sulfonamides, natural phenolic compounds sourced from *Origanum dictamnus* L. and *Thymus vulgaris* L have also emerged as β-CA inhibitors. Xanthomicrol and rosmarinic acid demonstrated submicromolar inhibition constants against MgCA [113]. However, despite favorable enzymatic potency, these compounds failed to inhibit *M. furfur* growth in vitro, again underscoring the disconnect that can occur between target engagement and whole-cell activity, likely due to permeability barriers [113]. Despite substantial biochemical characterization, a major limitation in this field remains the absence of cocrystal structures of *Malassezia* β-CAs complexed with inhibitors. Although sequence analysis and biochemical studies confirm the presence of the conserved Cys-His-Cys zinc-binding motif typical of fungal β-CAs, the lack of structural data constrains structure-based drug design and precise mapping of ligand-enzyme interactions [108, 114]. Future crystallographic studies will be essential to rationally optimize inhibitor selectivity, potency and the identification of key hydrophobic and hydrogen-bonding interactions within the active site.

### Agents targeting *Malassezia* lipases

*Malassezia* species rely on secreted lipases to hydrolyze host sebum triglycerides, releasing free fatty acids that serve as nutrients and contribute to scalp irritation, inflammation, and flaking. *Malassezia globosa* lipase-1 (SMG1) is central to this lipid-utilization pathway and represents a druggable antifungal target [115]. Targeting these lipases is, therefore, considered a promising antifungal strategy, as inhibiting SMG1 or related enzymes may both impair nutrient acquisition and reduce the release of pro-inflammatory fatty acids that exacerbate scalp irritation and flaking.

The metabolites from the fruit peels of *Punica granatum* L., rhizomes of *Glycyrrhiza glabra* L., and barks of *Cinnamomum zeylanicum* Blume were identified by GC–MS analysis and further evaluated by molecular docking to evaluate putative interactions with lipase-1 from *Malassezia globosa.* Table 3 reports the main analytes found in the extracts and the minimum binding energy of selected phytochemicals against SMG1 lipase. No in vitro validation was performed to corroborate in silico results [116].

**Table 3:** Main constituents identified by GC–MS and minimum binding energy (MBE) of selected phytochemicals against SMG1 lipase [116]

Compound	%Area	MBE (kcal/mol)
*G. glabra (ethyl acetate extract)*		
Glabridin	16.38%	− 5.13
3-Phenyl-2-propenal	8.07%	NA
Eugenol	4.64%	− 4.98
4'-*O*-methylglabridin	4.10%	− 4.88
Hispaglabridin A	2.67%	NA
Stigmast-5-en-3-ol	2.49%	− 6.9
Stigmasta-3,5-dien-7-one	2.39%	− 6.5
Glabrol	2.07%	− 10.12
*cis*-Dimethyl morpholine	2.54%	NA
Guanosine	2.42%	NA
*P. granatum (methanol extract)*		
Hydroxymethylfurfural	60.17%	NA
Stigmast-5-en-3-ol	6.65%	− 6.9
3,5-Dihydroxy-6-methyl-2,3-dihydropyran-4-one	5.11%	NA
1,2,3-Benzenetriol	3.53%	NA
1,6-Anhydro-α-D-glucopyranose	3.51%	NA
*C. zeylanicum* (*SFE-CO*_2_* extract)*		
Cinnamaldehyde	75.58%	− 4.96
α-Muurolene	4.41%	− 4.65
Caffeic acid	3.80%	NA
α-Copaene	2.18%	− 4.42
Calamenene	1.71%	− 5.75
*Trans*-β-caryophyllene	1.13%	− 4.37

NA, not available (compounds were not selected for molecular docking)

Four analytes showed better predicted affinity toward lipase-1 than the reference inhibitor RHC-80267 (− 5.83 kcal/mol), namely 22,23-dibromostigmast-5-en-3-yl acetate (DBSA; − 11.04 kcal/mol), glabrol (− 10.12 kcal/mol), stigmasta-5,3-dien-7-one (− 6.50 kcal/mol), and stigmast-5-en-3-ol (β-sitosterol) (-6.29 kcal/mol). Among these, only glabrol formed hydrogen bonds, interacting with Thr101, Gln282, and Val293, whereas the other three ligands did not form hydrogen bonds and were stabilized predominantly through hydrophobic interactions within the binding cavity. For comparison, the reference lipase inhibitor RHC-80267 formed hydrogen bonds with Thr101 and Leu103, indicating a somewhat different interaction pattern within the binding pocket.

The docking results were further supported by in vitro antifungal evaluation of the four best-scoring compounds identified in the docking study against *Malassezia furfur* using the agar well diffusion method. Among the tested compounds, glabrol produced the largest inhibition zone (20 mm), followed by DBSA (18 mm), while β-sitosterol and stigmasta-5,3-dien-7-one both produced inhibition zones of 16 mm. The reference inhibitor RHC-80267 showed a slightly smaller inhibition zone (15 mm). A similar trend was observed in the MIC assay, where glabrol exhibited the lowest MIC value (70 µg/mL), indicating the strongest antifungal activity. In comparison, DBSA showed a MIC of 150 µg/mL, whereas stigmasta-5,3-dien-7-one and β-sitosterol each showed MIC values of 160 µg/mL. The reference compound RHC-80267 displayed an intermediate MIC value (100 µg/mL). Overall, the biological results were broadly consistent with the docking analysis, particularly in the case of glabrol, which combined strong predicted binding with the most pronounced antifungal activity in vitro.

**Fig. 6:**
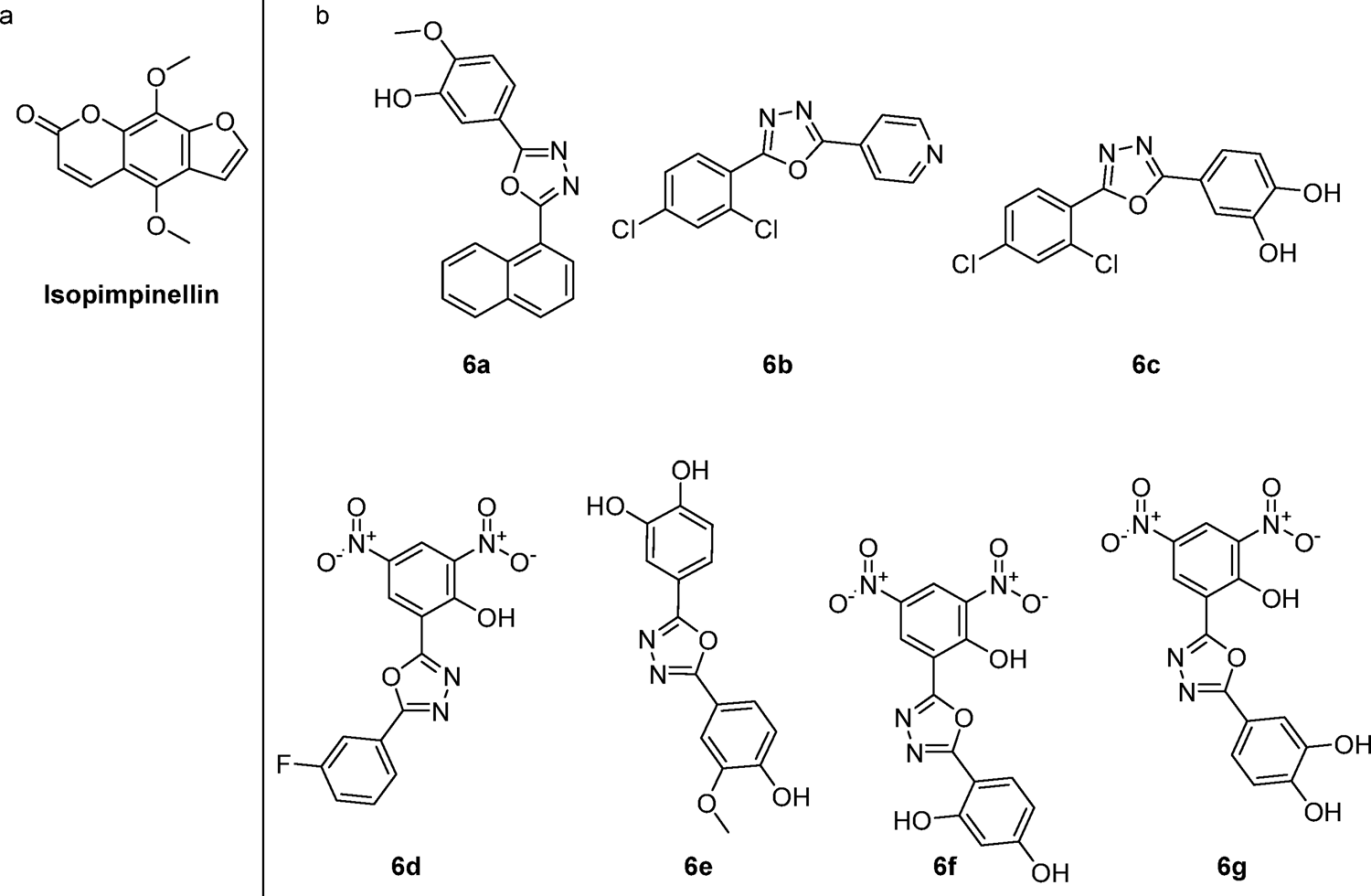
**a** Structure of isopimpinellin; **b** oxadiazole derivatives selected after molecular docking on lipase and evaluated against *M. furfur* [118]

Vishnupriya et al. conducted in silico analysis by molecular docking to evaluate protein–ligand interactions between the constituents of *Limonia acidissima* L. and lipase-1 from *Malassezia globosa.* The study evaluated 11 molecules: bergapten, dictamine, isoimperatonin, isopimpinellin, marmesin, marmin, osthol, psoralen, saponarin, umbrelliferone, and xanthoxol. All molecules showed lower atomic contact energies compared to standards ketoconazole (− 323.36 kcal/mol) and embelin (− 191.73 kcal/mol). Among these molecules, the binding between isopimpinellin (Fig. 6) and lipase-1 exhibited the highest atomic contact energy (− 162.32 kcal/mol), followed by bergapten (− 141.74 kcal/mol). Both compounds showed notable interactions with the Arg202 residue, which was also involved in the binding of five other evaluated molecules, suggesting a similar mode of interaction within the enzyme’s binding site. In contrast, the reference antifungal ketoconazole did not form any interactions with the enzyme’s residues, indicating a different binding pattern within the explored binding pocket [117].

Varma et al. performed molecular docking of 200 substituted diphenyl 1,3,4-oxadiazole derivatives to discover novel compounds with lipase inhibition capabilities (Fig. 6). The fifteen highest-scoring compounds interacted with Arg202, similar to the reference inhibitor RHC-80267, and also formed contacts with additional residues within the same binding pocket, including Arg236, Thr232, Ala237, Pro231, Leu270, Ser30, and His242. The best-ranked compound, 6a, with a docking score of -8.5 kcal/mol, formed four hydrogen bonds involving Thr232, Arg236, and Arg202. Compounds 6c, 6d, and 6e also established two to four hydrogen-bond interactions, while simultaneously interacting with a broader set of residues within the active site compared to the reference ligand.

Seven compounds (Fig. 6) were selected from the fifteen highest-scoring ligands based on their binding energies and were subsequently synthesized and evaluated for antifungal activity against *M. furfur*. Compound 6d exhibited the highest inhibition zone (33 mm vs 27 mm of ketoconazole), while compounds 6a, 6c, and 6g performed better than miconazole (20 mm). Furthermore, compound 6d showed anti-inflammatory activity and exhibited the highest percentage inhibition of bovine serum albumin (BSA) (86.54% at 100 μg/mL) compared to diclofenac (80.44%) [118].

The absence of any 3D cocrystal structures of mono- and diacylglycerol lipases complexed with inhibitor molecules in Protein Data Bank hinders a rational understanding of the structural determinants governing substrate specificity and inhibitors drug design. Xu et al. demonstrated that SMG1 possesses a catalytic triad composed of Ser171, Asp228, and His281. The nucleophilic residue Ser171 belongs to the conserved Gly-X-Ser-X-Gly motif and is positioned within the tight turn connecting the C-terminus of the β4 strand and the α3 helix, where it remains buried and inaccessible to solvent. The other two catalytic residues, Asp228 and His281, occupy canonical positions and orientations typical of the α/β-hydrolase fold [119]. Further investigation by Lan et al. on a similar mono- and diacylglycerol lipase from *Malassezia globosa* (*Mg*MDL2) confirmed the same catalytic triad. In this enzyme, however, the nucleophilic Ser171 lies at the apex of a “nucleophilic elbow” between strand β5 and helix 5. The catalytic His281, located within a ten-residue loop behind strand β10, is oriented toward the nucleophile [120]. During hydrolysis of the mono- and diacylglycerols, formation of the tetrahedral intermediate generates an oxyanion that is stabilized by the oxyanion hole, predicted in both SMG1 and *Mg*MDL2 to involve the backbone carbonyl oxygen atoms of Leu172 and Thr101. Two bulky residues, Trp229 and Phe278, situated adjacent to the catalytic triad, together with the extended hinge region of the open lid, appear to impose steric constraints that hinder triglyceride binding and contribute to the substrate specificity toward mono- and diacylglycerols of these enzymes. These distinctive structural characteristics of SMG1 and *Mg*MDL2 provide valuable insights into the structure of the catalytic site, the hydrolysis mechanism and offer potential guidance for the rational design of novel inhibitors.

Despite these advances, most studies on *Malassezia* lipase inhibitors remain limited to in silico analyses or early in vitro assays, and no validated clinical candidates have emerged so far. The lack of inhibitor-bound crystal structures significantly restricts structure-based drug design, and the physiological redundancy of lipases within *Malassezia* may complicate selective targeting. Future efforts should focus on resolving enzyme-inhibitor complexes, validating hits in biologically relevant models, and assessing whether lipase inhibition can translate into meaningful clinical improvement in dandruff and seborrheic dermatitis.

### Peptides

Antifungal peptides are comprised of short amino acid sequences, mostly cationic or amphipathic, that either occur naturally (such as bacteriocins) or are synthetic and usually more stable analogs of natural peptides. Antifungal peptides disrupt fungal cells through membrane permeabilization, disruption of membrane potential, induction of reactive oxygen species, and interference with intracellular targets. The multi-modal mechanisms make antifungal peptides promising alternatives to conventional antifungals because they are highly selective and less likely to drive the development of drug resistance [121]. In the context of *Malassezia*, several peptides, including lactoferrin-derived fragments, cathelicidin analogues, and synthetic variants engineered for improved stability, have demonstrated measurable in vitro inhibitory activity. Their predominantly membrane-targeting mode of action reduces the likelihood of resistance development and may potentiate the activity of existing antifungal agents.

Van Eijk et al. synthesized antimicrobial peptides derived from cathelicidin and evaluated the antifungal activity of peptides against strains of *Malassezia furfur*. The peptides included the patented scaffold RRWVQRWIRRWR as core sequence of amino acids. Peptides CR-172 and CR-184 exhibited inhibition of growth in range 0.3–5 µM over 47 h for DTO 383-D9 strain. For DTO 383-D8 strain, CR-172 showed inhibition only at 5 µM, while the activity of CR-184 ranged from 1.3 to 5 µM [122].

**Fig. 7:**
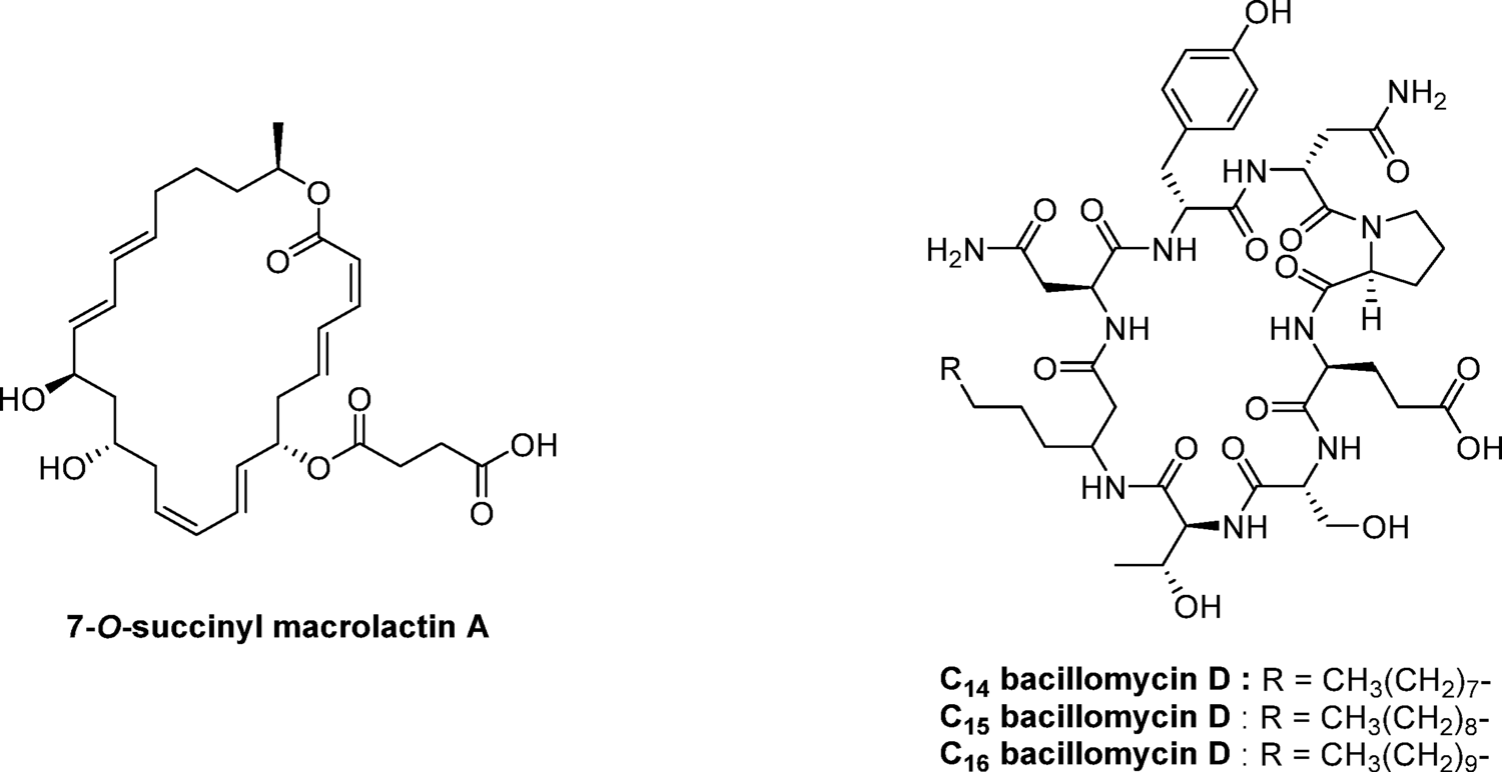
Molecular structures of antifungal metabolites [123]

Vairagkar et al. investigated metabolites isolated from the fermentation of *Bacillus amyloliquefaciens* for antifungal activity against strains of *Malassezia globosa* and *Malassezia furfur.* Reversed phase HPLC coupled with mass spectrometry revealed 7-*O*-succinyl macrolactin A, C14 bacillomycin D, C15 bacillomycin D, and C16 bacillomycin D (Fig. 7) as main compounds in the extracted fraction. The enriched extract with these metabolites showed between 7–8 mm diameter of inhibition, compared to 12 ± 0.42 mm for ketoconazole. Time-kill assay showed dose-dependent inhibition up to 5 min for 20 mg/mL [123].

Brouwer et al. reported the biological activity of a peptide derived from human lactoferrin against 30 *Malassezia furfur* strains. Peptide hLF(1–11), with sequence GRRRRSVQWCA, exhibited inhibition within 25–75 µg/mL (mean MIC = 42.53 µg/mL). Furthermore, the combination of hLF(1–11) with fluconazole or amphotericin B was evaluated. Fluconazole and hLF(1–11) showed synergistic effects for 21 strains (FIC value < 1), additive effects for 4 strains (FIC value = 1) and no significant effects for 5 strains (FIC value > 1). Similarly, amphotericin B and hLF(1–11) showed synergistic effects for 27 strains, additive effects for 2 strains, and no significant effects for only 1 strain [124].

Finally, Satanin 1, a Scarabaeidae cecropin from *O. curvicornis* and *D. satanas*, was evaluated in vitro against *Malassezia* reference strains (*M. furfur*, *M. pachydermatis*, *M. sympodialis*), showing MIC ranging from 12.5 to 50 µg/mL, with no correlation found between Satanin 1 MIC values and those of fluconazole or amphotericin B, used as reference drugs. In particular, Satanin 1 was particularly effective (MIC of 12.5 g/mL) against some clinical isolates, like *M. sympodialis* 1DA and *M. furfur* 959, which were resistant to both amphotericin B and fluconazole. Transmission Electron Microscopy (TEM) revealed that Satanin 1-exposed yeasts had a thin cell surface and light, non-uniform cytoplasmic content, suggesting disintegration and cell necrosis, potentially including intracellular leakage [125]. Despite these encouraging findings, the clinical translation of antifungal peptides remains limited. Challenges include susceptibility to proteolytic degradation, potential cytotoxicity at higher concentrations, and the difficulty of achieving sustained delivery through topical formulations. Further studies are required to improve peptide stability, define structure–activity relationships, and develop optimized delivery systems suitable for cutaneous application.

### Miscellaneous compounds and formulations

The use of fatty acids derived from esters has been already explored against *Malassezia* species [126], but the strong odor of optimal concentrations for therapeutic applications limits their adoption in cosmetic formulations. Koch et al. optimized the efficacy of fatty acid esters by evaluating derivatives of octanoic acid and undec-10-enoic acid against *M. furfur*, *M. globosa*, *M. restricta*, and *M. sympodialis*. The best performing derivatives at the agar dilution test were monoesters with glycerol or propane-1,3-diol, however their activity was less effective than climbazole and piroctone olamine, used as references (Table 4). Furthermore, combination of 2,3-dihydroxypropyloctanoate and 3-hydroxypropylundec-10-enoate at 2:1 ratio achieved synergistic effect (synergy index < 0.6 using Kull’s equation). The tested fatty acid esters effectively addressed the odor problem [127].

**Table 4:**
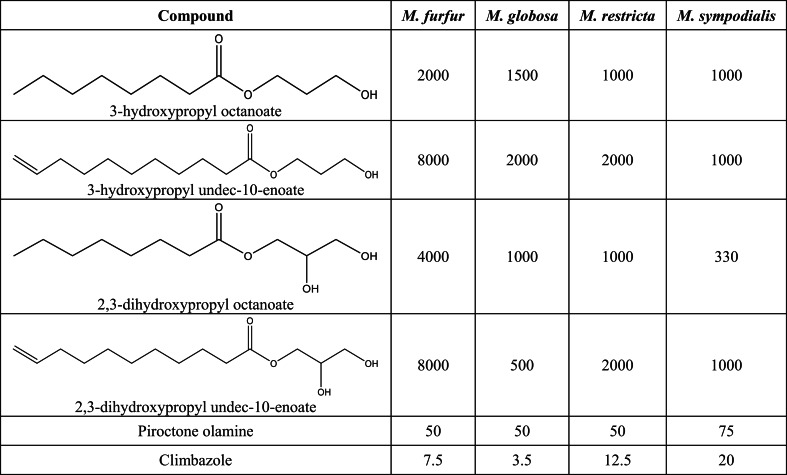
Minimal inhibitory concentrations (mean values, in ppm) of fatty acid esters with the highest activity [127]

**Fig. 8:**
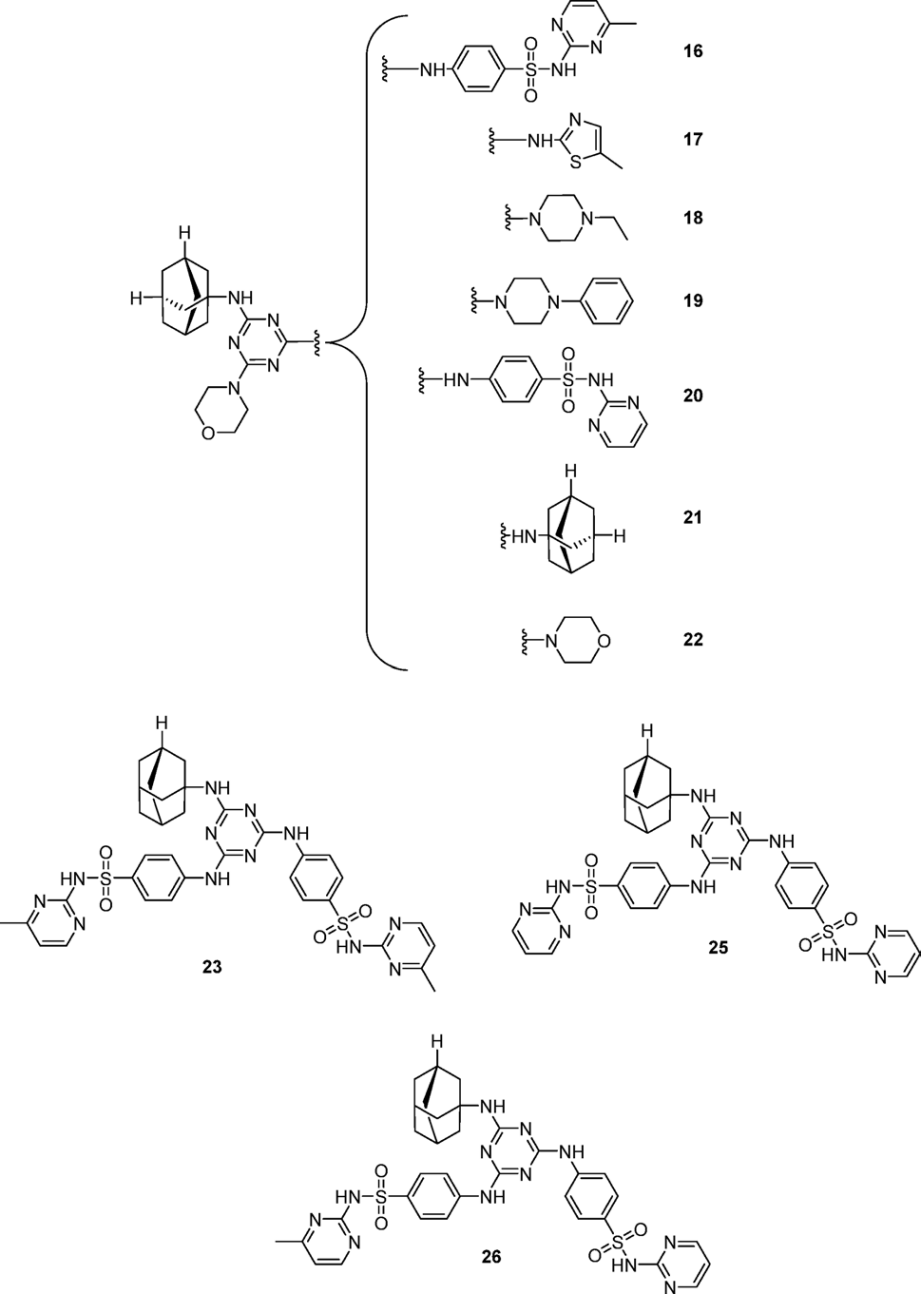
Triazine structures [128]

Kumawat et al. reported the synthesis of substituted triazine analogues (Fig. 8) and the biological evaluation of the compounds on *Malassezia furfur*. Compounds 16–22 shared both morpholine and 1-adamantylamine scaffolds. Structure activity relationships of compounds 16–22 showed that sulfadiazine (20) exhibited the best activity of the series, followed by *N*-ethyl piperazine (18), 2-amino-5-methylthiazole (17), 1-adamantylamine (21), *N*-phenylpiperazine (19), morpholine (22), and sulfamerazine (16). Compounds 23, 25, and 26 maintained the 1-adamantylamine scaffold. Compounds 23 and 25 possessed double sulfamerazine and sulfadiazine, respectively. However, their activity was similar to ketoconazole, but lower than most analogues of triazine. Interestingly, compound 26 with one sulfamerazine and one sulfadiazine performed better than compound 20. Overall, compound 26 exhibited the best MIC and IC_50_ values compared to ketoconazole and other analogues of triazine (Table 5) [128].

**Table 5:** Antifungal activity of triazine analogues and reference drug Ketoconazole, sorted from the best to the worst [128]

Compound	MIC (µg/mL)	IC_50_ (µg/mL)
26	8.13 ± 0.27	37.66 ± 0.57
20	9.34 ± 0.24	43.66 ± 0.57
18	12.21 ± 0.25	63.66 ± 0.57
17	13.23 ± 0.22	64.33 ± 0.47
21	15.31 ± 0.22	65.66 ± 0.47
19	17.42 ± 0.27	67.33 ± 0.47
25	19.22 ± 0.26	69.66 ± 0.47
Ketoconazole	19.26 ± 0.25	69.66 ± 0.57
23	20.46 ± 0.22	70.66 ± 0.47
22	26.41 ± 0.23	77.66 ± 0.57
16	35.32 ± 0.24	85.66 ± 0.57

Neaz et al. developed nanostructured lipid carriers made of climbazole, a commonly used antifungal agent, and zinc or selenium ions coupled with combinations of glycine (Gly) or pyroglutamic acid (L-PCA) with salicylic acid (L^1^) or glycolic acid (L^2^). Using well diffusion method, selenium complexes performed slightly better compared to climbazole alone, while zinc complexes exhibited slightly reduced inhibition zones compared to climbazole alone (Table 6). Moreover, in vivo treatment on volunteers showed reduction in scale and weight of dandruff [129].

**Table 6:** Antimicrobial activity of nanostructured lipid carriers (NLCs) and reference climbazole [129]

Preparation/compound	DOI (mm)
Climbazole	35 ± 0.04
[Se (L^1^) (Gly)]/Climbazole NLC	37 ± 0.08
[Se (L^2^) (Gly)]/Climbazole NLC	36.2 ± 0.04
[Zn (L^1^) (L-PCA)]/Climbazole NLC	33.4 ± 0.05
[Zn (L^2^) (L-PCA)]/Climbazole NLC	33.3 ± 0.04

DOI: diameter of inhibition

Finally, Ivanova and Buzova experimented with an enzymatic approach to target the colonization of *Malassezia* species and the production of dandruff. The synergistic combination of chitinase and chitosanase (100 U/g and 200 U/g, respectively) derived from *Aspergillus niger* resulted in the in vitro inhibition of *M. furfur*, *M. restricta*, and *M. globosa* by 98.38% at 0.25% w/w (*p* < 0.05), while total inhibition was observed at 0.5% w/w. A clinical study (18 volunteers) based on quantitative PCR demonstrated that the 0.25% w/w solution of chitinase and chitosanase effectively degraded cell walls of *Malassezia* fungi after 3 h of scalp treatment, thus decreasing DNA and RNA levels (2.4- and 2.4-fold change for *M. furfur* and 1.9- and 4.6-fold change for *M. restricta*, respectively) [130].

## Conclusions

Over the past few decades, the increasing incidence of *Malassezia*-related infections has highlighted the urgent need for medicinal chemists to develop new and effective strategies to control or eradicate this lipophilic yeast, particularly under conditions favoring its overgrowth. The current therapeutic arsenal, largely based on traditional azole antifungals, is often inadequate to prevent recurrence and cross-resistance. Consequently, recent research has focused on innovative strategies, including the design of *Malassezia* carbonic anhydrase inhibitors, lipase inhibitors, silver nanoparticles, and natural compounds, as well as other novel approaches targeting *Malassezia* species. Although *Malassezia* infections are not typically life-threatening, their prevalence in both humans and animals is rising, often leading to chronic or recurrent conditions influenced by host factors. Furthermore, growing evidence indicates a direct interaction between fungal components and the host immune system. Despite this clinical relevance, standardized susceptibility testing methods and validated clinical breakpoints for *Malassezia* are still lacking, complicating effective therapeutic management. Azoles remain the mainstay of treatment, yet they are hindered by side effects, resistance, and limited efficacy. In the absence of harmonized guidelines, there is a pressing need to identify new synthetic molecules and to better characterize natural compounds with potential activity against *Malassezia*. Among the emerging approaches, inhibitors of *M. globosa* carbonic anhydrase represent one of the most promising classes, supported by increasing in vitro and in vivo evidence of their antifungal potential. In contrast, lipase inhibitors are still in the early stages of exploration, with only a few molecules synthesized and evaluated so far. Other compound classes remain poorly characterized regarding antifungal potency, cytotoxicity, selectivity, and pharmacokinetic properties. Nevertheless, the evidence supporting natural products remains preliminary, as most studies rely on non-standardized extracts and lack rigorous mechanistic or in vivo validation. Beyond enzyme inhibition, recent work highlights the potential of probiotics to modulate the skin microbiota, restore microbial balance, and indirectly suppress *Malassezia* growth through competitive exclusion and metabolite production. Peptide-based antifungals also represent a rapidly growing field. Naturally occurring and synthetic antimicrobial peptides demonstrate potent activity against *Malassezia* through membrane disruption and immune modulation, with minimal cytotoxicity. These molecules, alone or incorporated into nanocarriers, may serve as effective components of new topical formulations.

## Data Availability

No datasets were generated or analysed during the current study.
